# Successful Outcomes, Fulfilment, and Positive Affect: Establishing Facets of Eustress Across Real-Life Challenging Situations

**DOI:** 10.1007/s11205-026-03909-6

**Published:** 2026-08-07

**Authors:** Juliane Kloidt, Lawrence W. Barsalou

**Affiliations:** School of Psychology and Neuroscience, University of Glasgow, 62 Hillhead Street, Glasgow, G12 8QB UK

**Keywords:** Eustress, Challenge, Distress, Stress mindset, Situated assessment

## Abstract

Establishing the features associated with eustress—the positive experience of a challenging situation—is essential for understanding and fostering eustress effectively in applied settings. Using the Situated Assessment Method, we conducted a pre-registered study in which 251 UK adults evaluated eustress in 20 real-life challenging situations (e.g., “Give a public speech in front of a large audience”). Participants also evaluated nine processes that potentially influence eustress in these situations, taken from the *C*omprehensive *H*ierarchical construct of *E*ustress, CHE (e.g., successful outcomes, fulfilment, positive affect). Finally, in a multilevel design, participants completed unsituated trait measures for eustress, distress, and stress mindset. Reliable individual differences were observed in average eustress judgments across situations (a trait-level measure), along with substantial situation effects and a large individual by situation interaction. The nine influential processes correlated strongly with eustress across situations, indicating high construct and content validity (explaining a median variance of 83% in individual-level regressions). Individual eustress averaged across situations correlated with trait eustress and trait stress mindset but was unrelated to trait distress. Exploratory factor analyses on situated and trait measures derived from the CHE model revealed a dominant general factor of eustress, accompanied by modest multidimensional structure. These findings align with existing theories of stress, contrast different types of eustress measurement, and provide rich descriptive profiles for personalized context-sensitive interventions to foster eustress when desirable.

**Supplementary Information:**

The online version contains supplementary material available at 10.1007/s11205-026-03909-6.

## Introduction

Eustress is a positive stress response that emerges from transactions between an individual and their immediate environment (cf. Lazarus & Folkman, 1984, 1987). Individuals experience eustress in various ways that can be cognitive (e.g., insightful appraisals, meaning), affective (e.g., fulfilment, excitement), and behavioral (e.g., coping, engagement), together impacting on the individual’s wellbeing, productivity, learning, and personal growth (Kloidt & Barsalou, 2024; Lazarus & Folkman, 1984; Simmons & Nelson, 2007). Eustress emerges from situations that are appraised as challenging, with most research to-date focusing on professional settings in corporations (Cavanaugh et al., 2000), social services (Rodríguez et al., 2013), and universities (Gibbons et al., 2009; O’Sullivan, 2011). By focusing on positive experiences in challenging situations (e.g., being excited about giving a public speech), eustress remains conceptually distinct from wellbeing, which typically occurs in non-challenging settings (e.g., being excited about relaxing at home). Nevertheless, the two constructs appear closely related (Kloidt & Barsalou, 2024).

Although eustress has often been conceptualized as a transactional process (cf. Lazarus & Folkman, 1987), relatively little research has addressed how individuals experience eustress across different areas of life and the underlying processes related to it. Understanding the processes that influence positive experiences of stress in real-world settings is crucial to inform contextualized interventions for pivoting from distress to eustress, such as interventions for producing stress optimization (Crum et al., 2020). The primary aim of this study was therefore to measure how UK adults experience eustress and its underlying processes across real-life challenging situations. We employed the Situated Assessment Method (SAM^2^; Dutriaux et al., 2023) to quantify self-reported eustress across individuals and challenging situations, and to identify key processes, such as successful outcomes, fulfilment, and positive affect, that influence it.

### The Situated Assessment Method (SAM^2^)

To understand a behavior in real-world situations, SAM^2^ proposes that researchers should measure the behavior in situations where it occurs, instead of ignoring situations or abstracting over them to simply measure traits. The few eustress instruments that exist typically adopt an *unsituated* approach, using decontextualized items to measure the trait of eustress, such as “In general, how often do you feel motivated by your stress?” (O’Sullivan, 2011). Certainly, these instruments add to our understanding of eustress as a theoretical construct and its general distribution across populations. Answering these items, however, can be difficult because individuals must abstract over situations and establish general impressions of how much they agree with general statements about eustress. Individuals may instead rely on intuitive theories and/or the availability heuristic (sampling a few salient situations) to arrive at their judgment, potentially leading to unrepresentative or inaccurate responses (Ajzen, 1977; Gelman & Legare, 2011; Tversky & Kahneman, 1973).

A large and well-established literature demonstrates that individual traits vary substantially across situations (Bandura, 1978; Cervone et al., 2001; Mischel & Shoda, 1995). For example, an individual may regularly experience eustress when performing their favorite piece of music or when presenting projects at work but rarely experience eustress when resolving family conflicts. For this reason, SAM^2^ assesses a target behaviour in specific situations where it is likely to occur. By doing so, trait-level eustress emerges as an individual’s average eustress across situations, while state-level eustress is captured as it varies in the individual across situations. Consistent with Fleeson and Jayawickreme’s (2021) *Whole Trait Theory*, capturing both trait-level and situation-level eustress in an individual is essential to understanding the expression of eustress in their life.

Additionally, different individuals may respond differently to the same situations, resulting in an individual by situation interaction (Dutriaux et al., 2023). Whereas one individual may experience eustress mostly in situations where a goal is pursued actively (e.g., presenting a work project), another individual may experience eustress mostly when engaging with the moment (e.g., listening to a favorite piece of music). As many accounts have suggested, the widely varying cognitive-affective-behavioral systems that develop in different individuals over the lifespan are responsible for these interactions (Bandura, 1978; Fleeson & Jayawickreme, 2025; Mischel & Shoda, 1995).

SAM^2^ further proposes that it is useful to assess processes in situations that are likely to influence the target behavior (Dutriaux et al., 2023). Whereas feeling engaged and fulfilled may be the main source of eustress when listening to music, experiencing productivity and successful goal achievement may drive eustress during a project presentation. For these reasons, SAM^2^ not only assesses a target behaviour across situations but also assesses processes likely to influence it. Empirical literatures related to the target behaviour are typically used to select the potentially important processes assessed. In previous work, SAM^2^ instruments have successfully established processes related to the performance of good and bad habits (Dutriaux et al., 2023), water drinking (Rodger et al., 2024), trichotillomania (Taylor Browne Lūka et al., 2024), and climate anxiety (Hill-Harding et al., 2024). Typically, these processes explain about 70% of the variance in an individual’s target behaviour.

In summary, SAM^2^ collects measurements on two dimensions of situatedness: (1) relevant situations where the target behavior may occur and (2) situational processes likely to influence the behavior in these situations. Thus, to implement a SAM^2^ instrument for the target behaviour of eustress, we first identified a set of real-world challenging situations and then identified processes in the literature that potentially influence eustress in them. Each phase of developing the SAM^2^ instrument is described next in turn.

#### Establishing Challenging Situations where Eustress Occurs

The SAM^2^ approach assesses a target behavior in individuals using a standardized set of situations. Strengths of this approach over other situated methods include that SAM^2^ can be performed efficiently in a single session and that individuals evaluate the same set of situations, such that a common standard of measurement underlies assessment (for a detailed discussion, see Dutriaux et al., 2023).

In this study, we developed a comprehensive yet diverse set of 20 situations that represent different intensities and types of challenge in the UK context (Table 1). These 20 situations were developed and evaluated previously in a related study with 81 UK adults (Kloidt et al., 2025), with the average challenge judgments ranging from low to high across situations, making this an appropriately diverse sample of situations for the study here. The 20 situations further differed in their associations between challenge and its underlying processes (e.g., intrinsic difficulty, anticipated loss, anticipated gain), indicating that different situations captured different types of challenges. Because the 20 situations represented different intensities and types of challenge, they should capture diverse manifestations of eustress with unrestricted variance. As Dutriaux et al. (2023) describe, presenting these situations to participants is likely to activate specific situational memories from their daily life that they then evaluate when responding to survey items.

**Table 1:** The 20 situations sampled from Kloidt et al. (2025)

Identifier	Situation
S01 – Decent signal	Try to find a decent Wi-Fi signal at a coffee shop
S02 – Hidden talent	Discover a hidden talent for photography and start sharing your work online
S03 – Long queue	Get stuck in a long queue at the post office
S04 – No GPS	Try to navigate a new city without a map or GPS
S05 – New recipe	Try to cook a new recipe with many steps for the first time
S06 – Squeaky door	Try to fix a stubborn squeaky door in your flat
S07 – Surprise visit	Handle a surprise visit from an old friend gracefully
S08 – Dream job	Start the process of finding and getting your dream job
S09 – Parking fine	Deal with a parking fine for overstaying in a parking spot
S10 – Customer service	Deal with a difficult customer service representative
S11 – Social media	Try to break free from your social media habits to focus on your mental health
S12 – Surprise party	Plan a surprise party for a loved one
S13 – Car accident	Handle the consequences of a minor car accident
S14 – Train schedule	Navigate a complex train schedule to get to a meeting on time
S15 – Work deal	Try to close a big deal at work and receive a significant bonus
S16 – Promotion	Taking on new responsibilities after receiving a promotion
S17 – Beloved pet	Saying goodbye to a beloved pet about to be put to sleep
S18 – Family argument	Get into a heated argument with a family member over a sensitive topic
S19 – Public speech	Give a public speech in front of a large audience
S20 – Marathon training	Develop and successfully execute a training plan to run your first marathon

#### Establishing Influential Processes of Eustress in these Situations

**Fig. 1:**
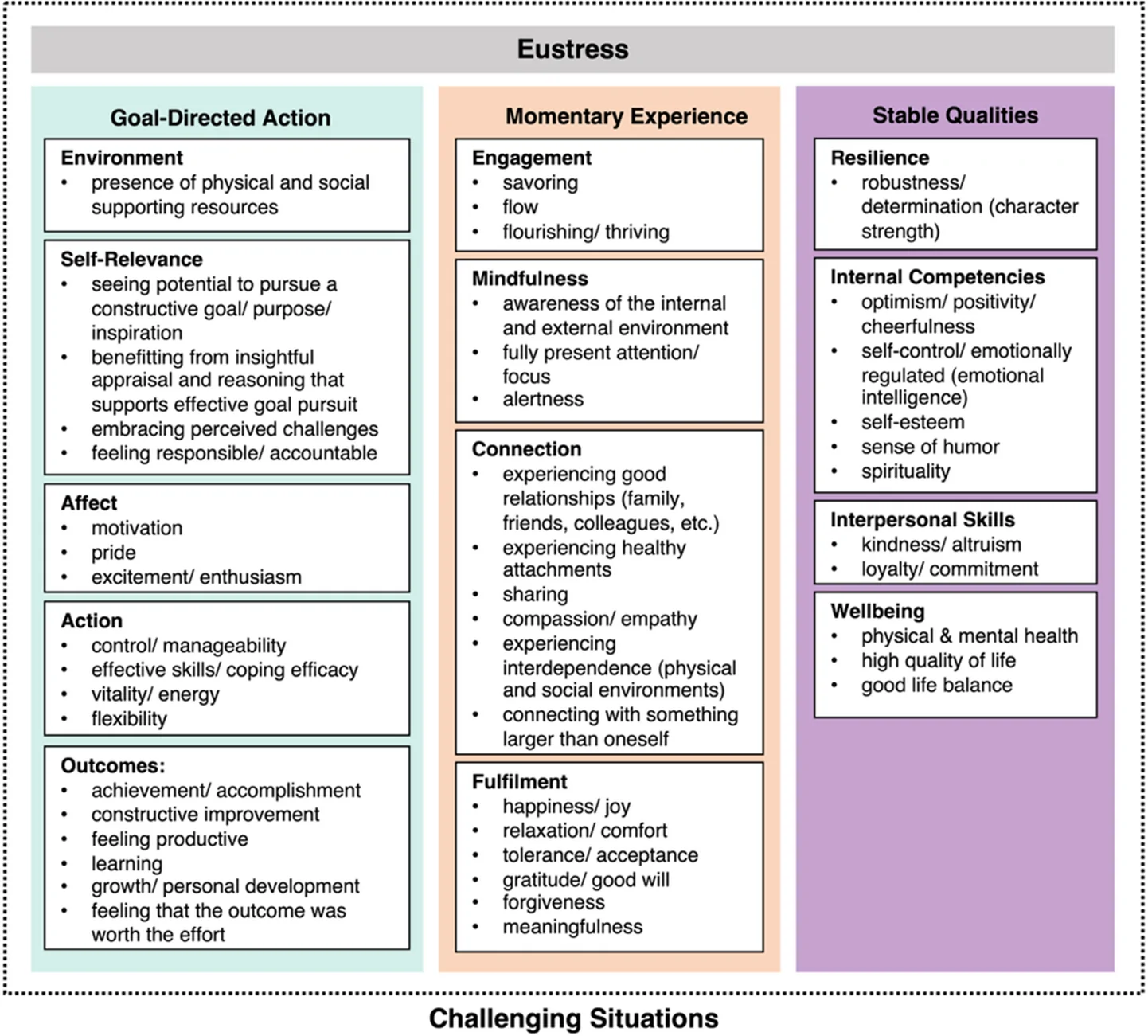
Comprehensive Hierarchical construct of Eustress (CHE). *Note*. CHE depicts three hierarchical levels of organization. The highest level are three eustress sources, depicted as colored columns (bolded headers). The sources contain 13 mid-level facets, illustrated as white boxes within each column (bolded box headers). The lowest level are 47 features organized into facet boxes (bullet points). Reproduced with permission from Kloidt and Barsalou (2024)

To establish a comprehensive set of processes that influence eustress in these situations, we drew on a recent theoretical model—the *C*omprehensive *H*ierarchical construct of *E*ustress (CHE; Kloidt & Barsalou, 2024)—because it integrates diverse eustress research from 80 articles and has been evaluated in a well-powered UK adult sample (Kloidt & Barsalou, 2025). Figure 1 presents the CHE model (reproduced from Kloidt & Barsalou, 2024).

According to CHE (and the diverse literatures on which it is based), individuals generate eustress in challenging situations from three main sources: successful goal-directed action, fulfilling momentary experience, and positive stable qualities. As Fig. 1 illustrates, the three high-level sources of eustress (colored columns) organize 13 distinct mid-level facets (white boxes) that cluster 47 conceptually related features (bullet points) at the lowest hierarchical level.

To establish influential processes for evaluation in our SAM^2^ instrument, we extracted CHE’s nine mid-level facets from the two sources of eustress in CHE most likely to influence eustress at the situational level: goal-directed action and momentary experience. We chose mid-level facets as they offered a balance between the high-level sources that would be too abstract to use and the low-level features that would be too detailed (and that would likely introduce redundancy in measurement). To create measurement items for the study here, we started with the facet labels in Fig. 1 and added details from their features to construct more explicit items that would be clear to participants in the context of the situations that they would be evaluating (e.g., affect → positive affect, action → coping and action, self-relevance → cognitive skills, outcomes → successful outcomes and personal growth).

We did not operationalize CHE’s third source of eustress—stable qualities—within the SAM^2^ instrument because, as the name suggests, it constitutes traits that remain relatively stable across challenging situations. As a result, they cannot be measured in a SAM^2^ instrument, which requires that test items vary across situations. Instead, we measured trait eustress separately from SAM^2^ at the individual level, using a subscale of the Comprehensive Hierarchical Eustress Review that assesses these traits (CHER-Qual; Kloidt & Barsalou, 2025). Later analyses will examine prediction at both the situational and individual levels. At the situational level, the nine facets for goal-directed action and momentary experience will be assessed. At the individual level, stable qualities will be assessed, along with other trait measures, including trait-level measures of goal-directed action and momentary experience, constructed by averaging all their facets across situations.

### Overview and Hypotheses

The current work aimed to investigate how individuals experience eustress and its underlying processes across real-life challenging situations where eustress is likely to occur. To do so, we developed a SAM^2^ instrument for assessing eustress in a situated manner. We asked a well-powered sample of UK adults to evaluate their eustress experiences across the diverse set of 20 everyday challenging situations presented in Table 1. We also asked participants to evaluate the same 20 situations on the 9 eustress facets (influential processes) that we sampled from the CHE model’s sources for goal-directed action and momentary experience. In addition to completing the SAM^2^ eustress instrument, participants completed three trait-level measures: the Comprehensive Hierarchical Eustress Review subscale for Stable Qualities (CHER-Qual; Kloidt & Barsalou, 2025), the Perceived Stress Scale (PSS-10; Cohen et al., 1983), and the Stress Mindset Measure (SMM; Crum et al., 2013).

This study was pre-registered on the Open Science Framework (https://osf.io/ydc25). To evaluate the SAM^2^ instrument for assessing eustress, we addressed the following hypotheses:

- *Hypothesis 1: Large reliable individual differences in eustress*

We expected that the average eustress judgments for each individual across situations would range over at least half of our 10-point rating scale from 2.5 to 7.5, indicating that participants exhibit large trait-level differences in eustress. We further expected that these individual-level eustress averages would exhibit satisfactory reliability (Cronbach’s alpha > 0.80).

- *Hypothesis 2a: Substantial situation effects*

We expected that a given participant would report high levels of eustress in some situations but little to no eustress in others. Based on previous studies, we expected that at least 33% of participants would range from 1 to 9 in their eustress judgments across situations on our 10-point rating scale, at least 67% of participants would range from 2 to 8, and at least 90% of participants would range from 3 to 7.

- *Hypothesis 2b: Substantial individual-situation interactions*

We expected that participants would differ considerably in how they experience eustress in the same situations, so that the intraclass correlation for agreement between participants would be low (*ICC2* < 0.40).

- *Hypothesis 3a: CHE’s facets for goal-directed action and momentary experience constitute situational processes related to eustress*

We expected that most (if not all) of CHE’s facets would be strongly related to eustress judgments across situations, indicating high construct validity for the SAM^2^ instrument. Specifically, we expected large positive correlations between eustress and each facet within a given individual (> 0.30; cf. Funder & Ozer, 2019; Gignac & Szodorai, 2016). As found previously in the literature, these facets should emerge as important situational processes associated with eustress.

- *Hypothesis 3b: CHE’s facets for goal-directed action and momentary experience explain individuals’ eustress in situations*

We predicted that CHE’s facets combined would explain high amounts of variance in individual-level regressions (> 50%). We thus expected that the influential processes explain eustress comprehensively, indicating high content validity of the SAM^2^ eustress instrument.

- *Hypothesis 4: Low correlations between situated eustress and unsituated measures*

We expected that the SAM^2^ measure for eustress at the individual level (an individual’s mean eustress across situations) would exhibit a small-to-medium, positive correlation (< 0.30) with the CHER-Qual sub-scale (i.e., trait eustress; Kloidt & Barsalou, 2025) and the Stress Mindset Measure (i.e., SMM; Crum et al., 2013). We also expected a small-to-medium, negative correlation (< |0.30|) between the SAM^2^ measure for eustress and the Perceived Stress Scale that measures trait distress (i.e., the PSS-10; Cohen et al., 1983).

#### Discovery

In a discovery analysis, we investigated how a variety of confirmatory factor models fit the individual-level data for facets of the three CHE sources of eustress (goal-directed action, momentary experience, stable qualities). We also conducted exploratory factor analyses to compare the best fitting exploratory model with the confirmatory models. Analogous to Hypothesis 4, we investigated whether factor scores from our discovery analysis would be correlated with the SAM^2^ measure for eustress.

## Methods

### Participants

Ethics approval was granted by the MVLS Ethics Committee at the University of Glasgow. We recruited a gender-balanced sample via the Prolific online platform that met the following inclusion criteria: UK residents, aged 18 to 80 years old, self-reported English fluency, and completion of at least 20 Prolific studies with 100% approval rate. As correlations stabilize at approximately 250 datapoints (Schönbrodt & Perugini, 2013), we recruited a slightly larger sample and inspected our data for mechanical or random responses. We excluded four participants for providing such responses, resulting in a final sample of 251 UK adults. Table 2 presents the sample’s demographic features, which approximated UK population demographics, except for slightly higher educational qualifications than the population average (Office for National Statistics, 2021, 2025).

### Design

This study used a multilevel design, with all participants at the individual level evaluating the same 20 situations at the situation level for eustress (the dependent variable) and for the nine facets that constitute processes potentially related to eustress. In addition, all participants completed three individual difference measures at the individual level.

**Table 2:** Participant demographics

Demographic	M (*SD*)	Range
Age	44.00 (14.43)	19 − 80
	Count	Proportion
Gender identity		
Man	125	.50
Woman	125	.50
Genderqueer/ Non-binary	1	.00
Annual income		
Below £10,000	29	.12
£10,001 – £20,000	42	.17
£20,001 – £30,000	74	.29
£30,001 – £40,000	42	.18
£40,001 – £50,000	34	.14
Above £50,001	30	.12
Years spent in formal education		
10 years or less (Level 1 or no qualification)	10	.04
11 – 12 years (Level 2)	35	.14
13 – 14 years (Level 3)	45	.18
15+ years (Level 4)	161	.64
	***Mdn (IQR)***	**Range**
Subjective social status	6 (4 – 7)	1 – 10

Years spent in formal education was measured on a discrete scale ranging from 0 to 20 + years and subsequently categorized into levels of qualification used by the UK Census: Level 1 (secondary school), Level 2 (secondary school completed), Level 3 (sixth form/ college completed), and Level 4 (at least one year of higher education/ university). Subjective social status was measured with the MacArthur Scale (Adler et al., 2000), where participants placed themselves on a 10-rung ladder representing where people stand in the United Kingdom (with higher rungs indicating higher subjective social status). *M* = mean; *SD* = standard deviation; *Mdn* = median; *IQR* = interquartile range. *N* = 251

### Materials

#### SAM^2^ Eustress Instrument

Following the SAM^2^ approach, the situated eustress instrument used the 20 situations in Table 1 together with the 10 judgment scales in Table 3 to assess eustress and related processes across situations. As described earlier, the situations were drawn from those developed by Kloidt and colleagues (2025), who evaluated the challenge levels and associated processes of these situations in UK adults. The judgment scales were motivated by the *C*omprehensive *H*ierarchical construct of *E*ustress (CHE; Kloidt & Barsalou, 2024) and the diverse literatures on which it is based.

#### Unsituated Individual Difference Measures

Three existing psychometric instruments were used to assess trait eustress, trait distress, and trait stress mindset: the Comprehensive Hierarchical Eustress Review subscale on Stable Qualities (CHER-Qual; Kloidt & Barsalou, 2025), the Perceived Stress Scale (PSS-10; Cohen et al., 1983), and the Stress Mindset Measure (SMM; Crum et al., 2013).

### Procedure

Following recruitment on Prolific, participants were referred to the Qualtrics platform, where they provided informed consent and completed the survey online (8 February 2025). All participants then completed the CHER-Qual followed by the PSS-10, the SAM^2^ eustress instrument, sociodemographic questions, and the SMM. As typically implemented, the order of CHER-Qual items was randomized, whereas the item order for the PSS-10 and SMM was fixed. For the SAM^2^ eustress instrument, all participants first completed the judgment block for eustress (dependent variable), with the order of the subsequent judgment blocks for the nine facets randomized. Within each judgment block, the order of situations was also randomized. Following study completion (*Mdn* = 31 min, *IQR* = 24–40 min), participants were debriefed, redirected to Prolific, and compensated with £4.50.

**Table 3:** Measures organized by eustress source, with descriptives and intraclass correlations

Measure / Item / Scale label	Source	*M* (*SD*)	Agreement (ICC2)	Coherence (ICC3)	Reliability (ICC3k)
Eustress	—	5.15 (2.97)	.27	.17	.81
How much eustress do you typically experience in this situation?					
(none / a little / moderate / strong / very strong)					
Environmental support	Goal	5.20 (2.74)	.17	.31	.90
How much support from your physical and social environment do you typically receive in this situation?					
(none / a little / moderate / strong / very strong)					
Cognitive skills	Goal	6.42 (2.60)	.25	.19	.83
How effectively can you use your cognitive skills to achieve goals that are relevant and important for you in this situation?					
(not at all / slightly / moderately / strongly / very strongly)					
Positive affect	Goal	5.58 (3.09)	.46	.21	.84
How much positive affect do you typically experience in this situation?					
(none / a little / moderate / strong / very strong)					
Coping and action	Goal	6.47 (2.53)	.13	.20	.83
How much can you cope and act effectively in this situation?					
(not at all / slightly / moderately / strongly / very strongly)					
Successful outcomes and personal growth	Goal	5.82 (2.92)	.39	.29	.89
How much do successful outcomes in this situation typically help you grow?					
(not at all / slightly / moderately / strongly / very strongly)					
Connection	Moment	4.72 (2.95)	.30	.24	.87
When in this situation, how connected do you typically feel with others?					
(not at all / slightly / moderately / strongly / very strongly)					
Mindfulness	Moment	6.32 (2.59)	.17	.28	.89
When in this situation, how mindful are you typically of what’s occurring?					
(not at all / slightly / moderately / strongly / very strongly)					
Fulfilment	Moment	5.69 (3.14)	.46	.21	.84
When in this situation, how much fulfilment do you typically experience?					
(none / a little / moderate / strong / very strong)					
Engagement	Moment	6.56 (2.72)	.27	.21	.84
When in this situation, how engaged are you typically of what’s occurring?					
(not at all / slightly / moderately / strongly / very strongly)					

The first measure is the dependent variable (eustress), followed by the nine facets extracted from the *C*omprehensive *H*ierarchical construct of *E*ustress (CHE; Kloidt & Barsalou, 2024). The name of each measure (bold) is followed by the item and scale labels. Participants rated all measures on continuous 0 to 10 self-report scales (one decimal place precision). The next column shows the CHE eustress source from which the facets were sampled: goal = goal-directed action, moment = momentary experience. The *ICC2* is the inter-rater agreement for each measure across the 20 situations, treating participants as random effects (Shrout & Fleiss, 1979). The *ICC3* is the coherence of the 20 situations in ordering participants, treating situations as fixed effects. The *ICC3k* is Cronbach’s alpha, capturing the measures’ test reliability in ordering participants by their aggregate ratings across situations. *M* = mean; *SD* = standard deviation. *N* = 251

### Analysis

Statistical analyses were performed in R (v4.3.2; Core Team, 2023) with the packages tidyverse (Wickham et al., 2019), psych (Revelle, 2024), and lavaan (Rosseel, 2012). Supplementary materials for the raw data, analysis files, and supplemental results files have been uploaded to the Open Science Framework including and can be accessed here: https://osf.io/62mn5/.

## Results

- **Hypothesis 1: Large reliable individual differences in eustress**

**Fig. 2:**
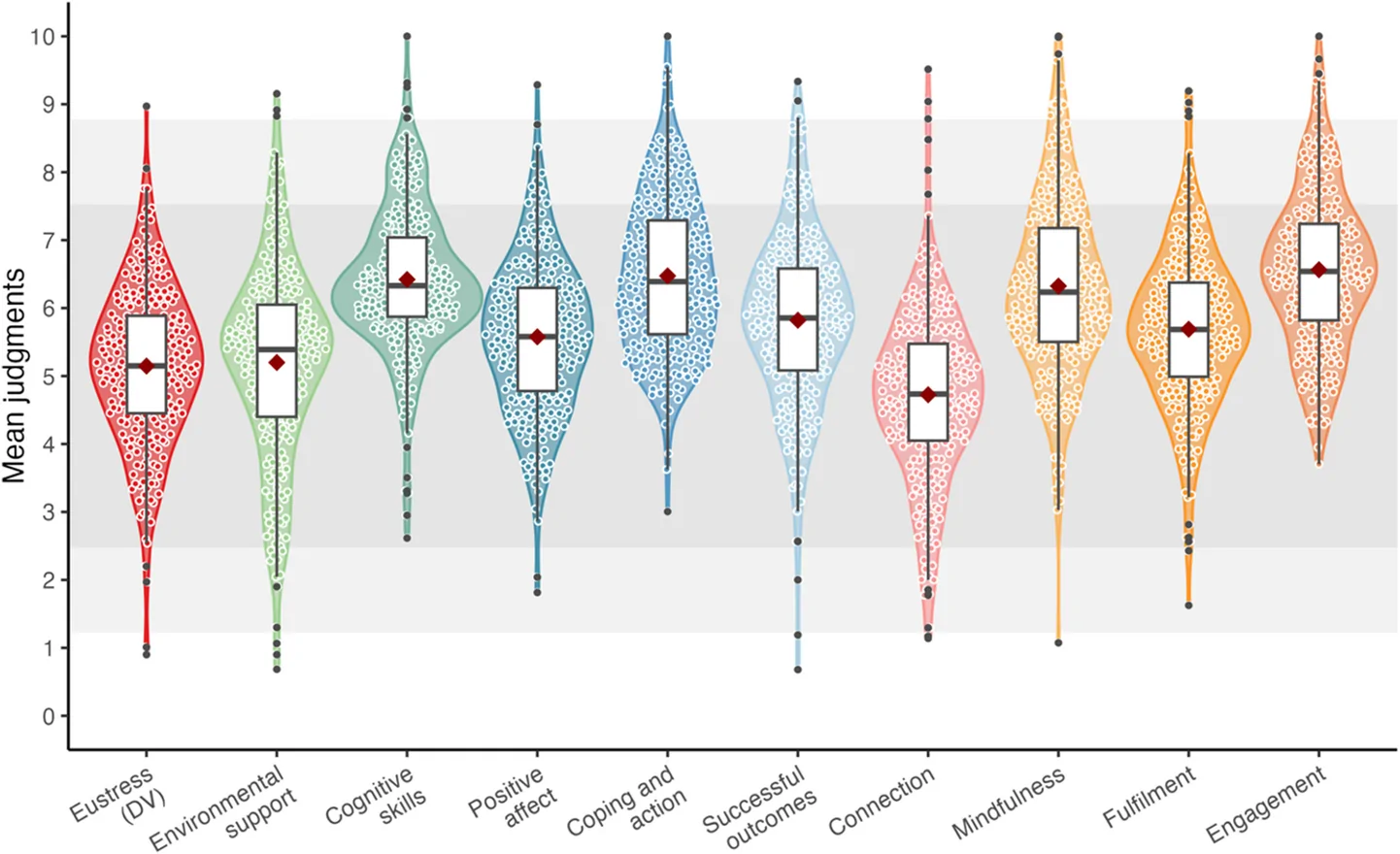
Participants’ mean judgments for eustress and its nine facets. *Note*. Violin plots show the distribution of participants’ judgments, averaged across 20 situations. Each dot represents the mean judgment from a single participant. Boxplots show the median for a measure and its interquartile range; superimposed diamonds depict the mean. The dark grey rectangle indicates 50% of the rating scale (2.5 − 7.5); the light grey rectangle indicates 75% of the rating scale (1.25 − 8.75). *N* = 251

We predicted large reliable individual differences in eustress when averaged across situations—what we will refer to from here on as *situated eustress*. Figure 2 shows each participant’s value for situated eustress (the dependent variable) and for its nine facets (influential processes) across the 20 challenging situations in the ten measures were assessed. As predicted, situated eustress showed large individual differences ranging from 0.90 to 8.97 (*M* = 5.15; *SD* = 2.97). Whereas some individuals experienced high levels of eustress across situations, others experienced little to no eustress.

As further predicted, Table 3 shows that mean judgments were reliable for eustress (Cronbach’s alpha as estimated with *ICC3k* = 0.81; Shrout & Fleiss, 1979), and also for its nine facets, where alpha ranged from 0.83 (cognitive skills, coping and action) to 0.90 (environmental support). Notably, Table 3 also shows that the 20 situations exhibited low coherence when ordering individuals according to their eustress judgments (*ICC3* = 0.17; Shrout & Fleiss, 1979) and to their 9 facets, ranging from 0.19 (cognitive skills) to 0.31 (environmental support). Thus, the SAM^2^ eustress instrument establishes satisfactory reliability through maximizing the coverage of the construct across situations (rather than via its saturation; Dutriaux et al., 2023). We therefore assessed reliability using Cronbach’s alpha, not with coefficient omega, which captures saturation instead.

- **Hypothesis 2a and 2b: Substantial situation effects and individual-situation interactions **

We predicted that individual eustress judgments would vary widely across situations, rather than exhibiting a constant trait level of eustress across them. We further predicted a large individual by situation interaction, as the level of eustress would depend not only on the situation but also on the individual.

**Fig. 3:**
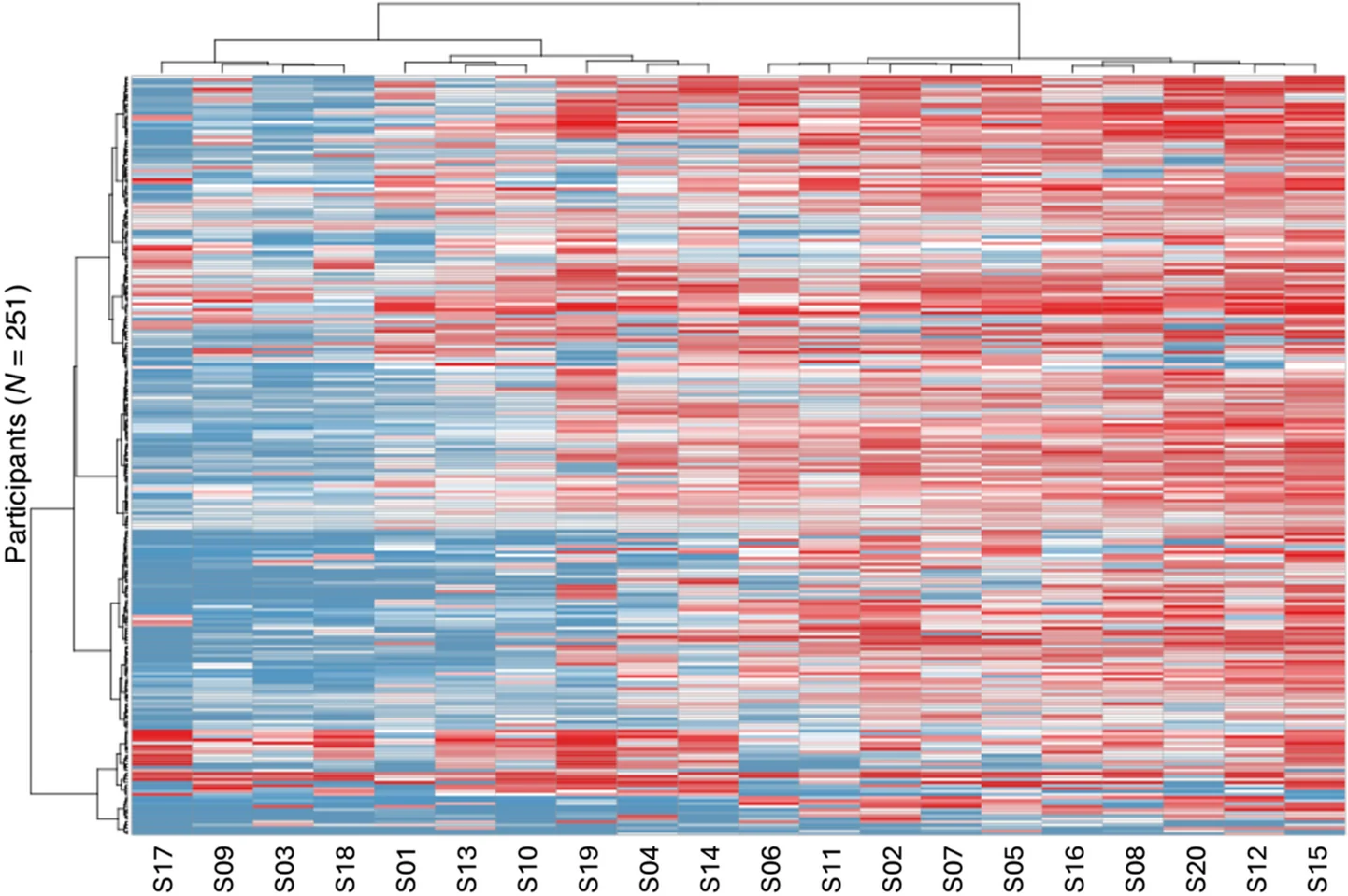
Heatmap of 5,020 eustress judgments. *Note*. Each row presents the 20 eustress judgments from a single participant. The index below each column indicates the corresponding situation in Table 1. As a cell becomes increasingly red, the eustress judgment increasingly approached 10 (on a 0 − 10 scale; Table 3). As a cell becomes increasingly blue, the eustress judgment increasingly approached 0. As a cell becomes increasingly white, the eustress judgment increasingly approached 5. Dendrograms from hierarchical Ward D clustering established groups of participants with similar eustress judgments across situations (left) and groups of situations with similar eustress judgments across participants (top). N = 251

Figure 3 visualizes the 5,020 eustress judgments obtained across individuals and situations as a heatmap. Each cell presents the eustress judgment from a specific participant (row) for a specific situation (column). Redder cells indicate higher eustress judgments and bluer cells indicate lower eustress judgments. The dendrograms cluster participants and situations that exhibit similar patterns of eustress judgments.

As illustrated by highly varying cell colors within each row of Fig. 3, participants’ eustress judgments varied substantially across situations. Consistent with Hypothesis 2a, 53% of participants (133 out of 251) ranged from 1 to 9 in their eustress judgments, 78% (197 out of 251) ranged from 2 to 8, and 94% (237 out of 251) ranged from 3 to 7. These findings support our prediction of substantial situation effects. Additionally, at the group level, the average eustress judgments across individuals varied widely from situation to situation, ranging from low (columns on the left) to high (columns on the right).

Figure 3 also demonstrates that individuals varied widely in the patterns of eustress they exhibited across the same 20 situations. This individual by situation interaction for eustress judgments is reflected in the differing color patterns between different rows of the heatmap (and clusters of rows). The intraclass correlation in Table 3 for agreement on eustress judgments (ICC2) quantifies the magnitude of this interaction, establishing the average correlation between participants in their eustress judgments across situations (Shrout & Fleiss, 1979). As predicted, the average correlation was low (ICC2 = 0.27), suggesting that individuals interacted with situations considerably

Finally, Fig. 3 visualizes the large differences in trait-level eustress between individuals shown earlier in Fig. 2, reflected here in the overall redness/blueness of a participant’s row. Supporting our predictions, the SAM^2^ eustress instrument captured substantial individual and situational variability, together with their interaction. As the other *ICC2* values in Table 3 indicate, large individual by situation interactions were also present for the nine CHE facets, ranging from 0.17 to 0.46.

- **Hypothesis 3a and 3b: CHE’s facets for goal-directed action and momentary experience predict and explain situated eustress**

**Fig. 4:**
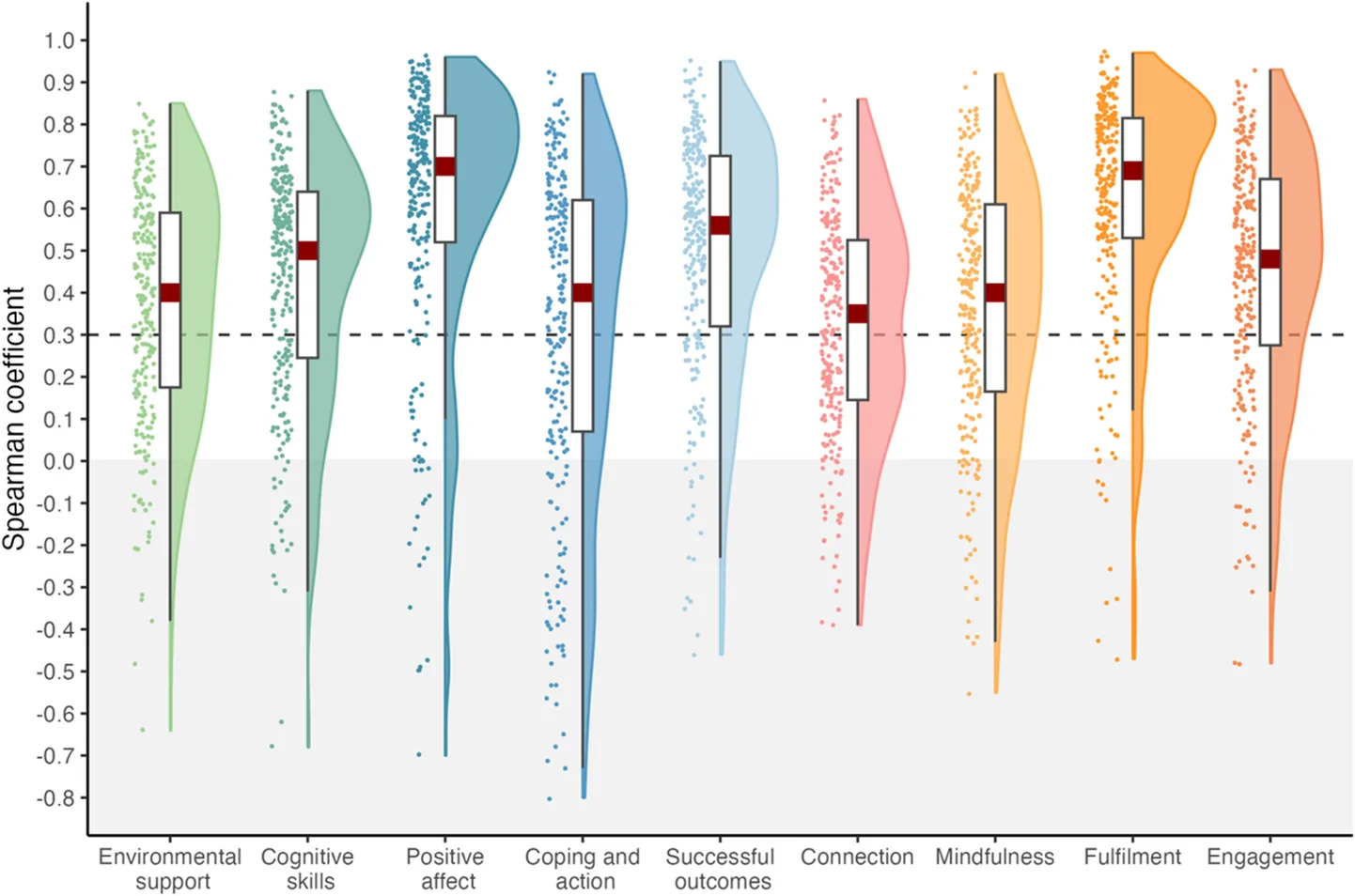
Predictive Spearman correlations of the nine CHE facets with eustress. *Note*. Scatter plots show the predictive correlation for a single participant, averaged across 20 situations. Half violin plots visualize the distribution of all participants. Boxplots show the group-level correlations, including the median (red segment) and the interquartile range (box). The dashed y-intercept depicts the cut-off for a large correlation (*ρ* = 0.30; cf. Funder & Ozer, 2019; Gignac & Szodorai, 2016), and the grey rectangle indicates negative correlations. *N* = 251

We expected that CHE’s facets would predict eustress judgments across situations, indicated by large positive correlation coefficients between these facets and situated eustress within a given individual (> 0.30; Funder & Ozer, 2019; Gignac & Szodorai, 2016). To assess Hypothesis 3a, we computed pairwise correlations between the judgments for each facet (influential process) and for eustress (the dependent variable) for one participant at a time. Based on our pre-registered assumption checks, we used Spearman correlations. To determine general patterns across our sample, we then computed the median of these pairwise correlations between eustress and each facet across all individuals. Figure 4 presents the pairwise correlations for each facet, showing the individual participant correlations as dots, and the median correlations as red segments within boxplots.

Figure 4 shows that, consistent with our prediction, all nine facets exhibited large median correlations with eustress (> 0.30). Across individuals in the sample, situated eustress correlated most strongly with positive affect (0.70), fulfilment (0.69) and successful outcomes (0.56), suggesting that experiencing eustress is closely related to feeling positive, fulfilled, and successful in challenging situations. In contrast, the weakest (yet still large) correlation with eustress was observed for connection (0.35), followed by environmental support, mindfulness, and coping and action (0.40 for all). These results perhaps reflect that not all eustress-inducing challenges offer meaningful opportunities for connecting and coping with the social and physical environment.

Similar to what we saw earlier in Fig. 2 for the trait level measures, individuals varied widely in how strongly each facet correlated with eustress, reflecting large individual differences. Interestingly, the spread of correlation coefficients was most pronounced for coping and action—the facet with the highest mean judgments in Fig. 2. This may indicate that although participants have numerous coping and acting strategies available, these strategies may not always be successful at producing eustress.

Having determined the prospective relationship between each facet and eustress, we next investigated how comprehensively the nine facets together explain each participant’s eustress judgments (Hypothesis 3b). To do so, we established the amount of variance in eustress judgments that a simple linear regression with fixed main effects for all facets explained for one participant at a time. The median individual variance explained across these individual regressions was 82.80% (*IQR* = 73.53% – 89.17%), indicating that the nine CHE facets capture situated eustress comprehensively.

The amount of variance explained was substantially higher on the individual level than on the group level, both for a simple group-level regression (*R*^*2*^ = 46.92%) and for a group-level linear mixed effects model with random intercepts for participants and situations (*R*^*2*^ = 52.79%). These differences are not surprising given the large individual variability in predictive strength that we saw in Fig. 4 that reduces prediction at the group level. Regardless, consistent with Hypotheses 3a and 3b, the individual-level correlations and regressions comprehensively predicted and explained eustress judgments, suggesting high construct and content validity of the SAM^2^ eustress instrument.

- **Hypothesis 4: Low correlations between situated eustress and trait-level measures**

We predicted small-to-medium correlations (< |0.30|; Funder & Ozer, 2019; Gignac & Szodorai, 2016) between situated eustress (each participant’s mean eustress across SAM^2^ situations) and the trait-level measures for eustress (CHER-Qual; Kloidt & Barsalou, 2025), stress mindset (SMM; Crum et al., 2013), and distress (PSS-10; Cohen et al., 1983). Again, we used Spearman correlations given that pairs of variables violated the necessary assumptions for Pearson tests. In addition to our confirmatory analyses, we explored the relationship between situated eustress and the situated CHE sources for goal-directed action and momentary experience. To do so, we computed the mean judgment for each participant across situations first for all facets belonging to goal-directed action (environmental support, cognitive skills, positive affect, coping and action, successful outcomes), and second, for momentary experience (connection, mindfulness, fulfilment, engagement).

As Table 4 illustrates, the pairwise correlation between situated and trait eustress (as assessed with the CHER-qual) was larger than predicted, *ρ* = 0.36, suggesting that dynamically changing states of eustress across situations showed a clear association with individuals’ general disposition for eustress as measured in an unsituated manner. Situated eustress, however, correlated more strongly with the CHE sources of goal-directed action (*ρ* = 0.51) and momentary experience (*ρ* = 0.52), perhaps because all three varied dynamically across situations. The pairwise Spearman correlation of situated eustress with stress mindset (the SMM) was 0.17, supporting our prediction that individuals’ eustress judgments in real-life situations exhibit a small association with their general belief that stress enhances outcomes across various areas of life. Similarly, the pairwise Spearman correlation between situated eustress and trait distress was quite small, –0.09, indicating that the two measures are weakly related at best. Notably, however, we observed a large negative correlation between trait eustress and trait distress (*ρ* = –0.57), suggesting that the absence of a correlation between situated eustress and PSS-10 distress may reflect differences in measurement (situated vs. unsituated) rather than construct independence (although they’re likely not inverses of each other; Kloidt & Barsalou, 2024).

**Table 4:** Spearman correlations with related constructs

	SAM^2^ Eustress	SAM^2^ Goal	SAM^2^ Moment	CHER-Qual	PSS-10
SAM^2^ Eustress	−				
SAM^2^ Goal	0.51	−			
SAM^2^ Experience	0.52	0.77	−		
CHER-Qual	0.36	0.50	0.46	−	
PSS-10	−0.09	−0.29	−0.20	−0.57	−
SMM	0.17	0.22	0.16	0.21	−0.17

Spearman correlations between participants’ situated judgments for eustress (SAM^2^ Eustress), goal-directed action (SAM^2^ Goal), momentary experience (SAM^2^ Experience), trait eustress (CHER-Qual), trait distress (PSS-10), and stress mindset (SMM). *N* = 251

### Discovery: Latent Structure of the CHE Facets

In a discovery analysis, we addressed whether facets from the same CHE source of eustress load onto a common general factor. Because Table 4 shows sizeable correlations between all three sources of CHE at the individual level, we not only included individual means from the nine situated CHE facets but also computed means for the four remaining CHE facets from the CHER-Qual subscale in Fig. 1 (resilience, internal competencies, interpersonal skills, wellbeing). For the situated facets, we calculated each participant’s mean judgment across situations. For the unsituated facets, we calculated each participant’s mean judgment across all CHER-Qual items belonging to the same facet (see Fig. 1 for the hierarchical structure).

Following an analysis pipeline developed in an earlier article that assessed the CHE model using an unsituated instrument, CHER (Kloidt & Barsalou, 2025), we performed a series of factor analyses on our facet data (fully reported in the Supplemental Materials here). We first assessed the three confirmatory models: (1) all facets load onto a general eustress factor (a unidimensional model), (2) the facets load onto three separate factors reflecting CHE’s three sources of eustress (a multidimensional model), and (3) each facet loads onto a general eustress factor and its respective source factor (a bifactor model).

All confirmatory factor solutions presented with non-acceptable model fit, indicating a misspecification of the factor loading structure (again, fully reported in the Supplemental Materials). We therefore conducted two exploratory factor analyses (EFAs) where facets could load freely onto three latent factors (resembling the multidimensional model) or onto a general factor and three orthogonal group factors (resembling the bifactor model). Compared to the confirmatory models, both EFAs presented with satisfactory model fit and adjusted yet meaningful facet loading structures. Here we focus on the best-fitting exploratory bifactor model and report the three-factor EFA in the Supplemental Materials.

**Fig. 5:**
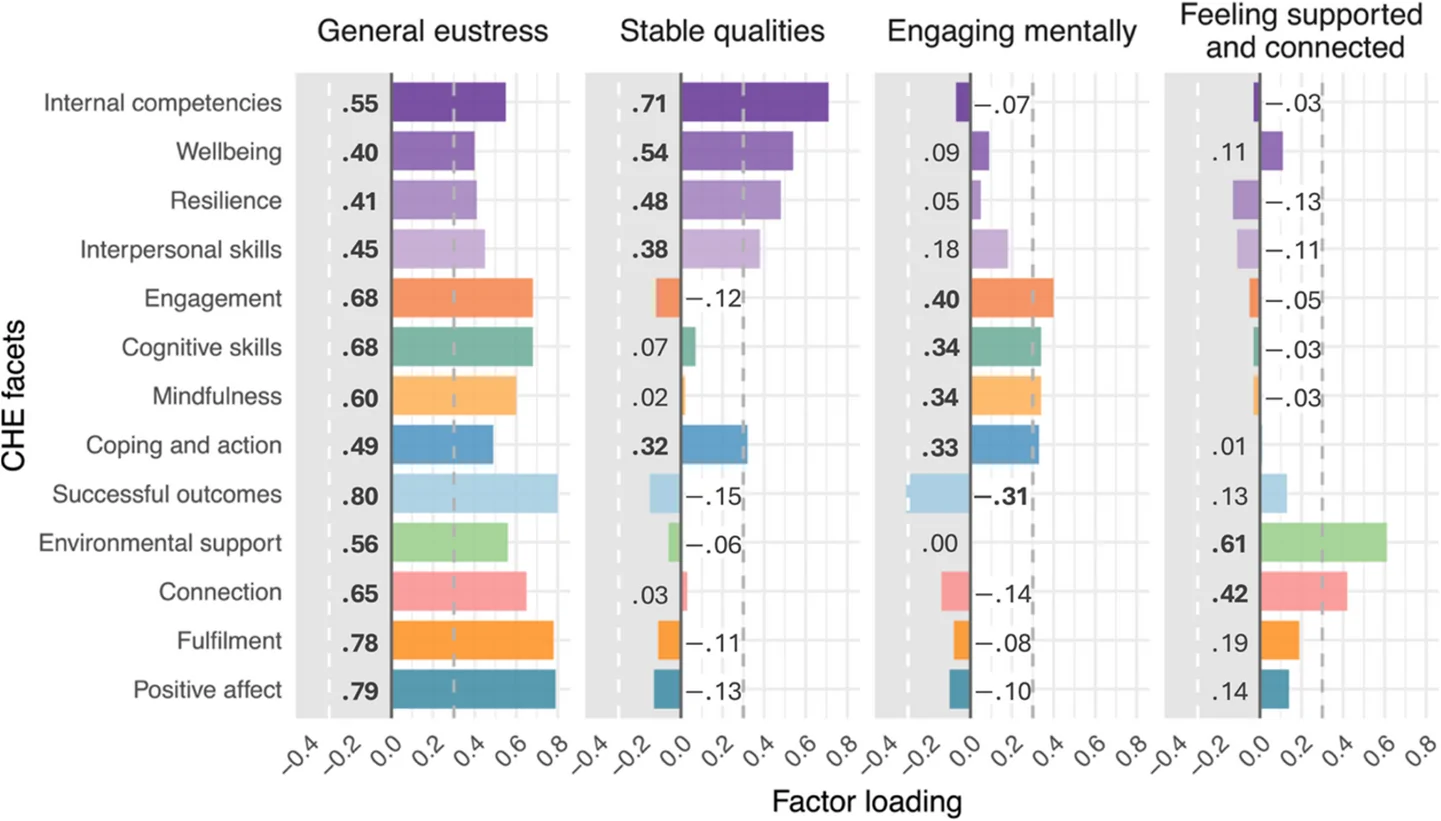
Factor loading plot for exploratory bifactor model. *Note*. The exploratory bifactor model with orthogonal factors accounted for 58.98% of total variance with most variance explained by “general eustress” (64.98%), followed by “stable qualities” (17.59%), “engaging mentally” (8.80%), and “feeling supported and connected” (8.62%). The facets are color-coded by CHE source: goal-directed action (green/blue), momentary experience (orange/pink), and stable qualities (purple). Factor loadings ≥ |0.30| are bolded and distinguished by dashed lines. Light gray rectangles distinguish negative factor loadings. *N* = 251

The exploratory bifactor model with orthogonal factors accounted for 58.98% of the shared variance and provided a satisfactory model fit, CFI = 0.971, TLI = 0.928, RMSEA (90% confidence interval) = 0.074 (0.053 − 0.096). As Fig. 5 illustrates, what we interpreted as a “general eustress” factor received meaningful loadings ≥ 0.40 from all 13 facets, including the strongest loadings for successful outcomes (0.80), positive affect (0.79), and fulfilment (0.78). The general factor accounted for 64.98% of the explained variance, demonstrating that the 13 CHE facets constitute a dominant general construct of eustress, similar to what we found in our earlier study with an unsituated eustress instrument (CHER; Kloidt & Barsalou, 2025). As can further be seen in Fig. 5, all nine facets for the goal-directed action and momentary experience sources in Fig. 1 loaded highest on the general factor, relative to the group factors. In contrast, only interpersonal skills from the stable qualities source loaded highest on the general factor. Again, though, all facets from all three sources clearly had a strong presence on the general eustress factor.

The three group factors captured residual variance in the facets after the general factor first accounted for common variance across them. The group factor that we interpreted as “stable qualities” closely resembles its respective CHE source with loadings > 0.30 from all its associated facets: internal competencies (0.71), wellbeing (0.54), resilience (0.48), and interpersonal skills (0.38). “Stable qualities” also received a sizeable cross-loading from coping and action, perhaps suggesting that individuals routinely apply coping skills in challenging situations in a stable manner. “Stable qualities” accounted for 17.59% of the explained variance, indicating that, in this model, the general factor benefits from an additional group factor for stable qualities.

The second group factor that we interpreted as “engaging mentally” received meaningful loadings > |0.30| from engagement (0.40), cognitive skills (0.34), mindfulness (0.34), coping and action (0.33), and successful outcomes (− 0.31), accounting for 8.80% of the explained variance. The third group factor that we interpreted as “feeling supported and connected” received sizeable loadings from environmental support (0.61) and connection (0.42), accounting for 8.62% of the explained variance.

Interestingly, the two group factors for engaging mentally and feeling supported/connected are similar to CHE’s sources of momentary experience and goal-directed action as follows: Whereas engaging mentally and momentary experience both refer to internal experiences of eustress, feeling supported/connected and goal-directed action both refer to external experiences. Although these two group factors are not identical to the corresponding CHE sources in Fig. 1, their similarity to them is consistent with CHE’s general structure of eustress.

Analogous to Hypothesis 4, we investigated whether factor scores from the exploratory bifactor model predicted situated eustress in our sample. Situated eustress and the general eustress factor exhibited a large Spearman correlation (*ρ* = 0.61). In contrast, situated eustress was uncorrelated with the group factor for stable qualities (*ρ* = 0.03), with the group factor for engaging mentally (*ρ* = −0.08), and with the group factor for feeling supported and connected (*ρ* = 0.10). These results are not surprising, given that group factors in a bifactor model capture residual variance between subgroups of facets after first contributing to common variance for the general factor. Additionally, each group factor only received meaningful loadings from a few facets; weakly-loading and cross-loading facets may have also created noise. Overall, though, the results from this discovery analyses suggest that facets from all sources of the CHE model constitute a dominant general eustress factor that strongly correlates with eustress in situations where it occurs.

## Discussion

We used the Situated Assessment Method (SAM^2^; Dutriaux et al., 2023) to investigate eustress in real-life challenging situations and to explore how a variety of situational and individual processes are related to it. Across the 20 situations assessed, a sample of UK adults exhibited moderate levels of eustress. As Hypothesis 1 predicted, trait-levels of eustress across situations varied substantially between individuals, indicating large individual differences (Fig. 2) that exhibited satisfactory reliability (Table 3).

In addition to individual differences, the SAM^2^ approach established large differences between situations (Fig. 3). As Hypothesis 2a predicted, some situations exhibited relatively high levels of eustress (e.g., S15 “Try to close a big deal at work and receive a significant bonus”), whereas others exhibited relatively low levels (e.g., S09 “Deal with a parking fine for overstaying in a parking spot”). As Hypothesis 2b predicted, a large individual by situation interaction emerged for eustress (Table 3), indicating that individuals experience eustress in the same 20 situations quite differently. These results demonstrate that both situation effects and individual by situation interactions are relevant for assessing eustress.

As Hypothesis 3a predicted, situated eustress judgments correlated strongly with the nine situated CHE facets for goal-directed action and momentary experience (Fig. 4), suggesting high construct validity for the SAM^2^ eustress instrument. At the group-level, eustress correlated most strongly with experiencing positive affect, fulfilment, and successful outcomes across challenging situations, with strong correlations also evident for the other six influential processes.

As Hypothesis 3b predicted, the nine situated CHE facets together explained high levels of variance in situated eustress judgments, suggesting high content validity of the SAM^2^ eustress instrument. In a group-level regressions, CHE’s facets explained 47% to 53% of the variance. At the individual level, the facets explained a substantially higher 83%, likely reflecting large individual differences that attenuated prediction at the group level. These results indicate that the SAM^2^ eustress instrument captures the content of eustress comprehensively.

The relations between situated eustress and unsituated individual difference measures only partially supported Hypothesis 4. Situated eustress and unsituated eustress (CHER-Qual; Kloidt & Barsalou, 2025) correlated more strongly than expected, providing evidence for the construct validity of both the SAM^2^ eustress instrument (Table 4) and the CHER instrument (Kloidt & Barsalou, 2025). As predicted, situated eustress exhibited a small positive correlation with stress mindset (SMM; Crum et al., 2013). Contrary to our expectations, situated eustress and trait distress (PSS-10; Cohen et al., 1983) were uncorrelated, likely reflecting differences in measurement (situated vs. unsituated) instead of conceptual independence.

Lastly, in a discovery analysis of the SAM^2^ measures at the individual level, we explored the factor structure of the nine situated facets from CHE’s sources for goal-directed action and momentary experience and the four unsituated facets for stable qualities (Fig. 1). In an exploratory bifactor analysis, a general latent factor emerged with loadings for all 13 CHE facets ≥ 0.40. This general eustress factor accounted for 65% of the explained variance and correlated strongly with situated eustress. Notably, all 9 facets for the eustress sources of goal-directed action and momentary experience loaded higher on the general eustress factor than on any of the more specific group factors. One facet from stable qualities—interpersonal skills—also loaded highest on it. Thus, all three of CHE’s eustress sources were strongly represented on the general eustress factor.

Three secondary group factors accompanied the general eustress factor in the bifactor model (Fig. 5). Notably, the group factor that we interpreted as “stable qualities” explained the most variance of the three group factors, indicating that stable qualities played an important secondary role in the structure of eustress, as captured in this analysis. Additionally, the other group factors that we interpreted as “engage mentally” and “feeling supported and connected” roughly parallel CHE’s sources for momentary experience and goal-directed action by aligning on internal versus external experience, respectively.

### Theoretical Implications

Our results demonstrate that experiences of eustress reflect contributions from individuals, situations, and interactions between individuals and situations (Fig. 3). Additionally, a variety of situational factors influence eustress as well, such as successful outcomes, fulfillment, and positive affect (Fig. 4). Thus, our findings closely align with the Transactional Model of stress and with Whole Trait Theory, suggesting that all types of stress responses (including eustress) reflect large individual by situation interactions (Lazarus & Folkman, 1984, 1987). Only focusing on individual-level trait measures of eustress masks considerable individual-specific variability at the situational level (Dutriaux et al., 2023; Fleeson & Jayawickreme, 2021). Conversely, because different individuals experience different patterns of eustress across the same situations, the situation alone is not the sole cause of eustress experience. Instead, each individual’s unique cognitive-affective-behavioural system contributes as well (Bandura, 1978; Cervone et al., 2001; Dutriaux et al., 2023; Fleeson & Jayawickreme, 2025; Mischel & Shoda, 1995).

A variety of results support the *C*omprehensive *H*ierarchical construct of *E*ustress (CHE; Kloidt & Barsalou, 2024). The finding that all nine influential processes predicted eustress at both individual and group levels confirms the importance of CHE’s two sources of state eustress (goal-directed action and momentary experience) from which they were extracted. As these results indicate, situational processes from the CHE model predict and explain dynamically changing states of eustress across real-world challenging situations. The additional finding that situated eustress correlated with the CHER-Qual scale indicates that trait-like dispositions in the CHE model exhibit meaningful associations with situated eustress as it manifests in goal-directed action and momentary experience. These results indicate that the CHE model captures eustress at both the state and trait levels, as Kloidt and Barsalou (2024) suggested.

Combining CHE’s 9 situated facets and 4 unsituated facets in an exploratory bifactor model revealed that a dominant general eustress factor meaningfully captured all 13 facets. The three group level factors that accompanied the general eustress factor—demonstrating modest multidimensional structure—aligned with the CHE model, with one reflecting stable qualities, one reflecting external experience (similar to goal-directed action in CHE), and one reflecting internal experience (similar to momentary experience in CHE).

The dominant presence of a general eustress factor here strongly mirrors the analogous finding of a dominant general factor in our unsituated assessment of eustress with the CHER instrument (Kloidt & Barsalou, 2025). Although multidimensional structure was modestly present in both assessments of eustress, a general eustress factor dominated each. Additionally, all three CHE sources for goal-directed behaviour, momentary experience, and stable qualities had a clear presence on each general factor (with minor differences between them). This common pattern suggests that the construct of eustress has a strong representation of the three CHE sources at its core. An important issue for future research is better understanding why these three sources merge together in this manner to form a general dimension of eustress.

### Measurement Implications

As for any other behavior, eustress can be operationalized in an unsituated study design—measuring traits—or in a situated study design—measuring dynamic states across situations, which can then be averaged to establish traits (Dutriaux et al., 2023; Fleeson & Jayawickreme, 2025). Whereas Kloidt and Barsalou (2025) examined the CHE model in an unsituated design (asking individuals to intuitively average their experiences across challenging situations in their lives), the current study examined eustress and its state-level processes in a situated design (asking individuals to evaluate eustress in a diverse set of real-life challenging situations).

Again, both types of study design suggest that eustress is best explained by a bifactor model with a dominant general factor and three complementary group-level factors. In both studies, the group factors aligned with the three sources of eustress in CHE but did not explain much additional variance beyond the general eustress factor.

This pattern indicates that, in both an unsituated and a situated study design, eustress can be assessed effectively with a single subscale that measures the general construct (similar to the assessment of general intelligence; Deary, 2012). If of interest, additional subscales that measure specific sources of eustress can also be included (similar to specific types of intelligence; Deary, 2012). Here, different subscales may be useful for different study designs: In the unsituated CHER instrument, the “goal-directed action” group factor explained the most additional item variance, and thus seems most relevant for complementing the general eustress factor that focused more on momentary engagement and stable qualities (Kloidt & Barsalou, 2025). In the situated SAM^2^ study design, the “stable qualities” group factor explained the most additional item variance, and thus, seems most relevant for complementing the general eustress factor that focused on state-level influences associated with goal-directed action and momentary experience.

The proposal that situated vs. unsituated measurements capture related but not the same information has been observed across various target behaviors (Dutriaux et al., 2023; Taylor Browne Lūka et al., 2024). Our findings further highlight the need for nuance when measuring a target behaviour in situated and unsituated manners, recognizing that complex interactions of multiple processes govern real world behavior and its assessment (Barsalou, 2019).

Although the specific situations and measures developed here could be used in future assessments of eustress, variants that use smaller numbers of situations and measures may be more efficient and yet equally effective. For discussion of approaches to adapting SAM^2^ instruments for specific applications, see Dutriaux et al. (2023) and Hill-Harding et al. (2024).

### Applied Implications

**Fig. 6:**
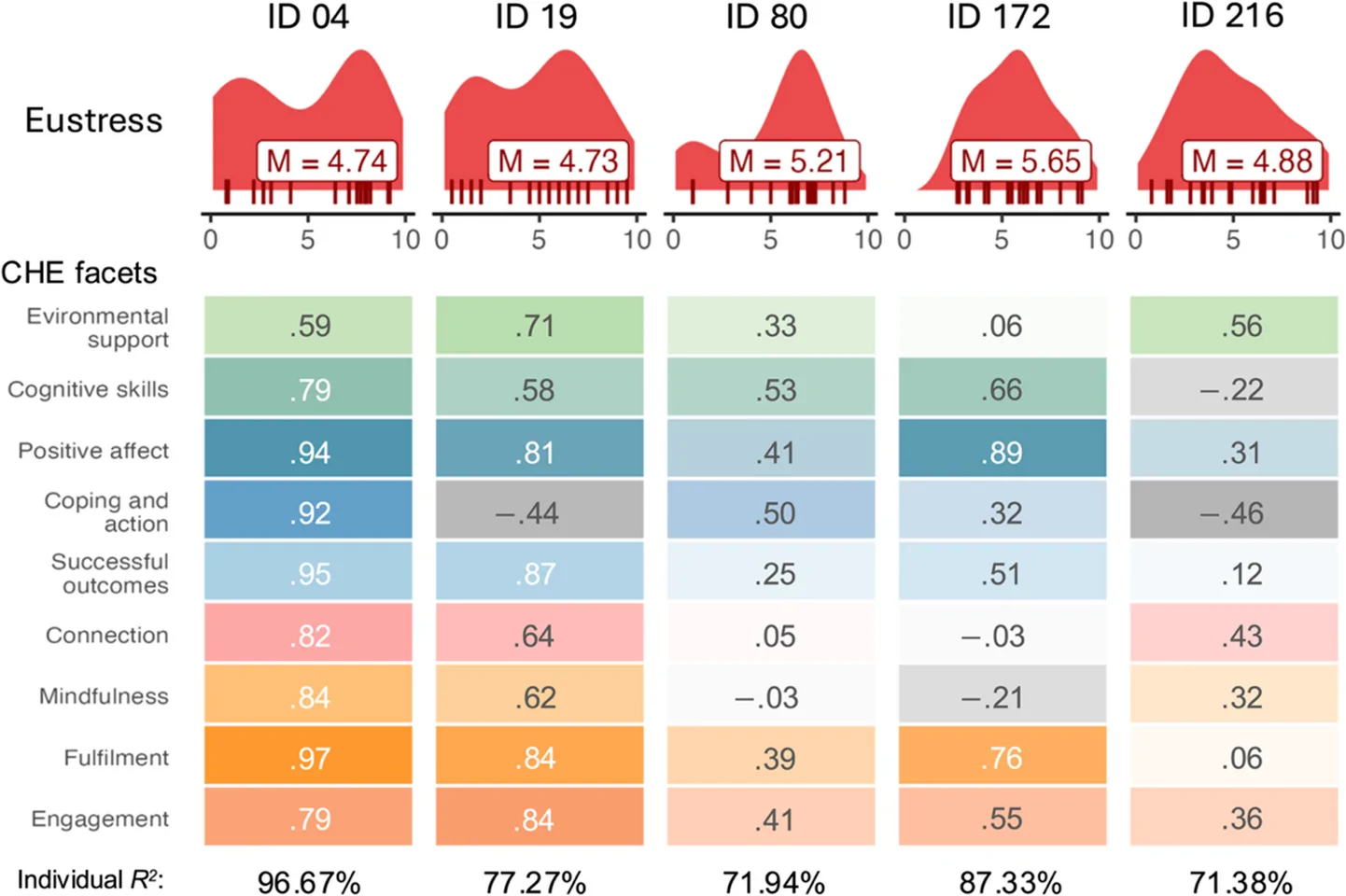
SAM^2^ predictive eustress profiles for five participants. *Note*. Each column presents the predictive profile of an individual participant. The upper panel presents the eustress judgments as distribution (density plot), situation-specific judgments (tile plots), and means across situations (superimposed M label). The lower panel illustrate the pairwise Spearman correlations between the participant’s eustress judgments and the nine CHE facets. The individual *R*^*2*^ shows the variance that the facets combined explain in eustress judgments of the individual. CHE = *C*omprehensive *H*ierarchical construct of *E*ustress; *ID* = identification number; *M* = mean; *R*^*2*^ = total variance explained

Individual-level prediction profiles from the SAM^2^ approach can be used to identify facets that most likely foster eustress in personalized interventions (Dutriaux et al., 2023). Figure 6 illustrates prediction profiles for five participants in the current study. For each participant, the upper panel presents their distribution of eustress judgments across the 20 situations (dark-red tiles), along with their general disposition for eustress overall (the M labels). The lower panel illustrates the pairwise Spearman correlations between each participant’s eustress judgments and the nine situated CHE facets. The individual R^2^ shows the variance that the facets combined explained in each individual’s eustress judgments across situations.

All participants in Fig. 6 present with moderate eustress levels that could be increased if desirable. If, for example, eustress is positively and strongly associated with all situated CHE facets (participant 4), then strategies to enhance any facet could help enhance eustress. If eustress exhibits different combinations of positive, negative, and negligible correlations with CHE facets (participants 19, 80, 172, 216), then strategies to enhance the facets with large positive correlations could be targeted specifically in an individualized intervention (e.g., environmental support for participant 216).

Another potential opportunity to explore is how eustress profiles could be used to implement stress optimization interventions in specific individuals (Crum et al., 2020). By examining an individual’s eustress profile, potential targets for changing their stress mindset may emerge. Being low on cognitive skills, for example, might suggest targeting an increase in self-efficacy, whereas being low on positive valence might suggest targeting an increase in positive reframing.

The SAM^2^ approach could also help identify challenging situations with the highest potential for increasing eustress (Dutriaux et al., 2023). A contextualised intervention could then identify what processes foster eustress for specific individuals in these situations (Craig et al., 2018). Coping and action, for instance, may be a highly useful skill in challenging situations that an individual has control over (e.g., S05 “Try to cook a new recipe with many steps for the first time”) but be less useful, and potentially frustrating, in less controllable situations (e.g., S07 “Handle a surprise visit from an old friend gracefully”). Predictive eustress profiles may offer insights into useful skills at the group level (Fig. 4) and at the individual level (Fig. 6).

### Limitations and Further Directions

A key limitation of our research approach is that the results are correlational and should not be used in isolation to inform causal explanations of eustress (Diener et al., 2022; Grosz et al., 2020). Specifically, our results do not provide insights into the mechanisms by which the nine situated CHE facets causally affect eustress, nor, in turn, how eustress causally affects these facets. Nevertheless, our results provide evidence that these facets (and their underlying sources) are likely relevant to a theoretical understanding of eustress. Researchers could therefore expand upon our findings by manipulating these facets and observing the effects, if any, that they have on eustress.

Another limitation of our research is that it is situated in the UK context, such that the situations we identified may not be comprehensive or relatable in other contexts. To the extent that different populations experience eustress in different contexts, the situations in Table 1 need to be adapted (Dutriaux et al., 2023).

The generalizability of our research is further limited by our non-probabilistic sample design (i.e., participants were recruited without information about selection probabilities; Heeringa et al., 2017). Because our measurement models are only partially independent from our sample, our results may not generalize fully towards the target population, even though the sample characteristics approximated UK adult population averages. Future research could therefore use SAM^2^ to identify relevant situations for other target populations, explore variations in eustress facets across these different contexts, and collect data from probabilistic samples allowing for more robust and generalizable inferences.

Another limitation of this research is the potential of measurement error in our eustress measures, given that it relied on self-report judgments. Participants may be unable to provide accurate self-reports about their eustress because they lack a detailed understanding of the construct and/or lack conscious access to relevant thoughts and feelings (Corneille & Gawronski, 2024). Whereas participants evaluated eustress and its nine facets here on quantitative scales, future research could gain further insights into individuals’ understandings of eustress using qualitative methods. Although our situated assessment aimed to help participants access relevant situation-specific memories from their daily life (cf. Dutriaux et al., 2023), future research could also measure eustress in real-time using ecological momentary assessments.

A final limitation is that our approach to eustress has been not informed by physiological stress responses or relevant neural systems. Because research often shows that psychological and physiological responses to stress are relatively disjointed, this may not be a critical issue (Becker et al., 2023; Epel et al., 2018). Because increasing work, however, establishes tighter couplings between psychological and biological stress responses, exploring the biological bases of eustress is becoming an increasingly important research direction (Betson et al., 2025; Crum et al., 2020; Sommerfeldt et al., 2019). An interesting possibility with respect to the work reported here is whether CHE’s three sources of eustress—goal-directed action, momentary experience, and stable qualities—are grounded in different biological processes, and similarly how a general eustress factor is grounded in the brain and body (Kloidt & Barsalou, 2024).

## Conclusion

We adopted a SAM^2^ approach to investigate complexities in eustress—the positive experience of a challenging situation—focusing on both individual and situational variability as underlying sources of it. Our results highlight substantial individual by situation interactions that align with transactional models of stress. Situational processes from the *C*omprehensive *H*ierarchical construct of *E*ustress (CHE) predicted individuals’ eustress judgments strongly across situations. Exploratory analyses suggest that situated eustress can be best measured as a dominant general factor, in the context of a weaker multidimensional structure. Additionally, rich descriptive profiles from our multilevel data can inform personalized context-sensitive interventions that foster eustress when desirable.

## Supplementary Information

Below is the link to the electronic supplementary material.

Supplementary Material 1.

## Data Availability

Data files and analysis scripts are publicly accessible on OSF at https://osf.io/62mn5/. All other materials used in the research are included in the manuscript and supplemental materials.

## Declarations

### Preregistration

This study was preregistered on OSF at https://osf.io/ydc25.

### Preprint

Kloidt, J. & Barsalou, L. W. (2025). Coping, fulfilment, and successful outcomes: Assessing sources of eustress across real-life challenging situations. PsyArxiv. 10.31234/osf.io/m8txb_v1.
