# Supplementary material for: Successful Outcomes, Fulfilment, and Positive Affect: Establishing Facets of Eustress Across Real-Life Challenging Situations
Source: Soc Indic Res. 2026 Aug 7;184(1):20. doi: 10.1007/s11205-026-03909-6 (PMC13451411; doi:10.1007/s11205-026-03909-6)
Supplement: Supplementary file 1 [file 11205_2026_3909_MOESM1_ESM.docx]

**Supplemental Materials**

**Successful outcomes, fulfilment, and positive affect:**

**Establishing facets of eustress across real-life challenging situations**

Juliane Kloidt

Lawrence Barsalou

School of Psychology and Neuroscience

University of Glasgow

24 July 2026

**Factor analysis pipeline**

In the main text of this article, we explored whether facets from the same CHE source of eustress (goal-directed action, momentary experience, and stable qualities) would load on a common factor. Because Table 4 in the main text exhibited sizeable correlations between all three sources of CHE, we not only included the nine situated CHE facets from the SAM^2^ instrument but also computed scores for the four remaining CHE facets from the CHER-Qual subscale (resilience, internal competencies, interpersonal skills, wellbeing). For the situated facets, we calculated each participant’s mean judgment across situations. For the unsituated facets, we calculated each participant’s mean judgment across all CHER-Qual items belonging to the same facet (see also Figure 1 in the main text).

To explore the latent structure of the 13 CHE facets, we first conducted three confirmatory factor analyses following an analysis pipeline developed in an earlier article that established the psychometric structure of eustress when being measured with the CHER instrument (Kloidt & Barsalou, 2025). The CHER instrument measures eustress with an *unsituated* instrument that includes CHE’s 47 low-level features as test items (see Figure 1 in the main text). In contrast, we here explored the latent structure of CHE’s 13 facets (not features), that were assessed with unsituated and situated measures. We opted for a facet-level measurement to strike a reasonable balance between comprehensively measuring eustress and avoiding potential redundancy of some features (as reported in Kloidt & Barsalou, 2025). We added situated measures to capture dynamic variations in the CHE facets that reflect state eustress. Thus, because our data captured the CHE model at a different hierarchical level with different measurement methods, we also conducted exploratory factor analyses in addition to Kloidt and Barsalou's (2025) confirmatory factor analyses.

To evaluate how well our data fit each model’s predicted structure, we report the following fit indices: comparative fit index (CFI), Tucker-Lewis index (TLI), root mean squared error of approximation (RMSEA), and Bayesian information criterion (BIC). For acceptable fit, CFI and TLI should be > .90 and RMSEA should be < .08 (Brown, 2015). Lower relative values for the BIC indicate more parsimonious solutions.

**Confirmatory factor analyses**

Following Kloidt and Barsalou's (2025) analysis pipeline, we tested the following confirmatory models. Model 1 employed a single latent factor to test if eustress is best understood as a simple unidimensional construct. Model 2 tested CHE’s multidimensional structure as a three-factor model reflecting CHE’s three sources of goal-directed action, momentary experience, and stable qualities. Model 3 tested the possibility that eustress reflects both a general eustress factor and three multidimensional factors, presented as a bifactor model.

***Model 1 (unidimensional)***

Our first confirmatory factor model consisted of one general factor, assuming that eustress is a unidimensional construct without any specificity that reflects multidimensionality. Thus, for Model 1, the 13 CHE facets loaded onto a single latent eustress factor (see Table SM-1). Model 1 only explained 39.09% of the total item variance and presented with non-acceptable model fit according to our absolute fit criteria, CFI = .728, TLI = .673, RMSEA (90% confidence interval) = .160 (.147−.174), and a relative model fit of BIC = 10,291. Whereas the 13 CHE facets exhibited a large mean factor loading of .61, the facets for stable qualities exhibited substantially smaller factor loadings (.41 – .54), indicating that trait eustress was not captured well by the single eustress factor. Our next analysis therefore assessed whether a more complex, multidimensional model could better represent the 13 CHE facets.

**Table SM-1**

*Model 1 (unidimensional)*

| **Source** | **Facet** | **Eustress *(𝛌)*** | ***𝛅*** |
| --- | --- | --- | --- |
| Moment | Fulfilment | **.80** | .36 |
| Goal | Positive affect | **.79** | .38 |
| Goal | Successful outcomes | **.76** | .42 |
| Moment | Connection | **.71** | .50 |
| Goal | Cognitive skills | **.67** | .55 |
| Goal | Environmental support | **.64** | .59 |
| Moment | Engagement | **.64** | .59 |
| Moment | Mindfulness | **.59** | .65 |
| Qual | Internal competencies | **.54** | .71 |
| Goal | Coping and action | **.51** | .74 |
| Qual | Interpersonal skills | **.45** | .80 |
| Qual | Wellbeing | **.44** | .80 |
| Qual | Resilience | **.41** | .84 |

*Note.* One-factor solution capturing the 13 CHE facets. Model 1 accounted for 39.09% of total variance. Factor scores ≥ .30 are bolded. Moment = momentary experience; Goal = goal-directed action; Qual = stable qualities. *λ* = factor loading; *δ* = uniqueness. *N* = 251

***Model 2 (correlated factors)***

The second confirmatory model assessed CHE’s structure in Figure 1 (main text), examining whether three factors reflecting goal-directed action, momentary experience, and stable qualities capture the aggregate measures of the 13 CHE facets effectively. As implemented by Kloidt and Barsalou (2025), facets could only load onto their respective factor, but inter-factor correlations were permitted to capture potential overlap between factors.

Compared to Model 1, Model 2 explained more total item variance (47.65%) and exhibited improved, yet still non-acceptable absolute model fit for CFI = .844, TLI = .804, and RMSEA (90% *CI*) = .124 (.110−.138) and improved relative model fit for BIC = 10,125. As illustrated by Table SM-2, the mean factor loadings were slightly higher for goal-directed action and momentary experience (.67 and .68 respectively) and substantially higher for stable qualities (.70), indicating that Model 2 captured state eustress and trait eustress.

Importantly, however, the inter-factor correlation between goal-directed action and momentary experience was > 1, suggesting an improper solution. We therefore next investigated whether highly overlapping factors indicate the presence of a general factor in addition to the three factors representing CHE’s high-level sources.

**Table SM-2**

*Model 2 (correlated factors)*

| **Source** | **Facet** | **Goal-directed**  **action *(𝛌)*** | **Momentary experience *(𝛌)*** | **Stable qualities *(𝛌)*** | ***𝛅*** |
| --- | --- | --- | --- | --- | --- |
| Goal | Positive affect | **.81** | – | – | .35 |
| Goal | Successful outcomes | **.78** | – | – | .39 |
| Goal | Cognitive skills | **.66** | – | – | .56 |
| Goal | Environmental support | **.64** | – | – | .59 |
| Goal | Coping and action | **.47** | – | – | .78 |
| Moment | Fulfilment | – | **.81** | – | .34 |
| Moment | Connection | – | **.72** | – | .49 |
| Moment | Engagement | – | **.63** | – | .60 |
| Moment | Mindfulness | – | **.56** | – | .68 |
| Qual | Interpersonal skills | – | – | **.88** | .23 |
| Qual | Wellbeing | – | – | **.69** | .52 |
| Qual | Resilience | – | – | **.62** | .61 |
| Qual | Internal competencies | – | – | **.59** | .65 |

*Note.* Confirmatory three-factor solution capturing the three sources of CHE that the 13 facets were extracted from. The source of goal-directed action comprised five facets; the sources of momentary experience and stable qualities comprised four facets each. Model 2 accounted for 47.65% of total variance with the factors contributing at the following proportions: goal-directed action explained 37.60%, momentary experience explained 30.37%, and stable qualities explained 32.03%. Factor scores ≥ .30 are bolded. Goal = goal-directed action; Moment = momentary experience; Qual = stable qualities. *λ* = factor loading; *δ* = uniqueness. *N* = 251

***Model 3 (bifactor)***

The final confirmatory model tested whether combining Models 1 and 2 would yield satisfactory model fit and conceptual interpretability. Model 3 therefore included the same three-factor structure as Model 2 but with an additional general eustress factor. All factors were orthogonal, ensuring high discriminant validity.

Compared to Models 1 and 2, the bifactor model explained the most total item variance (56.03%) and the best relative model fit, BIC = 10,081. Nevertheless, most of the absolute fit indices remained non-acceptable, CFI = .902, TLI = .853 and RMSEA (90% *CI*) = .108 (.092−.123). In Model 3, the general factor captured a dominant construct of eustress, presenting with the same mean factor loading as in Model 1 (.61) and accounting for 69.42% of explained variance. Compared to Model 2, the three group factors presented with lower absolute mean factor loadings and less variance accounted for: goal-directed action (*λ* = .29, R^2^ = 7.44%), momentary experience (*λ* = .29, R^2^ = 5.52%), and stable qualities (*λ* = .55, R^2^ = 17.61%). These results are not surprising given that group factor scores in a bifactor model represent residual variance between subgroups of facets after first contributing to common variance for the general factor.

We note that the group factor goal-directed action received a substantial negative loading from coping and action (–.51), undermining the model’s conceptual interpretability. The results from Model 3 therefore indicate that, although the bifactor model represented CHE’s facets better than Models 1 and 2, the confirmatory bifactor structure may still include misspecifications that could be resolved using less restrictive, exploratory modelling.

**Table SM-3**

*Model 3 (bifactor)*

| **Source** | **Facet** | **Eustress *(𝛌)*** | **Goal-directed action *(𝛌)*** | **Momentary experience *(𝛌)*** | **Stable qualities *(𝛌)*** | ***𝛅*** |
| --- | --- | --- | --- | --- | --- | --- |
| Goal | Coping and action | **.53** | **–.51** | – | – | .46 |
| Goal | Successful outcomes | **.78** | **.44** | – | – | .20 |
| Goal | Cognitive skills | **.68** | –.21 | – | – | .49 |
| Goal | Environmental support | **.64** | .16 | – | – | .56 |
| Goal | Positive affect | **.80** | .15 | – | – | .34 |
| Moment | Engagement | **.66** | – | **.41** | – | .39 |
| Moment | Mindfulness | **.59** | – | **.39** | – | .50 |
| Moment | Connection | **.72** | – | –.26 | – | .42 |
| Moment | Fulfilment | **.80** | – | –.10 | – | .34 |
| Qual | Internal competencies | **.50** | – | – | **.74** | .20 |
| Qual | Wellbeing | **.40** | – | – | **.56** | .53 |
| Qual | Resilience | **.37** | – | – | **.49** | .62 |
| Qual | Interpersonal skills | **.40** | – | – | **.41** | .67 |

*Note.* Confirmatory bifactor solution with orthogonal rotation modelling the 13 CHE facets and their three CHE sources. The general factor includes all CHE facets whereas the specific factors reflect CHE’s three sources of goal-directed action (five facets), momentary experience (four facets), and stable qualities (four facets). Model 3 accounted for 56.03% of total variance with the factors contributing at the following proportions: “general eustress” explained 69.42%; “stable qualities” explained 17.61%, “goal-directed action” 7.44%, and “momentary experience” 5.52%. Factor scores ≥ |.30| are bolded. Goal = goal-directed action; Moment = momentary experience; Qual = Stable qualities. *λ* = factor loading; *δ* = uniqueness. *N* = 251

**Exploratory models**

Because the confirmatory factor analysis pipeline from Kloidt and Barsalou (2025) did not identify a well-fitting and conceptually interpretable solution, we next considered data-driven models. We conducted exploratory factor analyses (EFAs) where facets could load freely onto three latent factors (resembling Model 2) or onto a general factor and three group factors (resembling Model 3). Because our models were exploratory, we did not restrict the facet loading structure, so that shared item variance could manifest as cross-loadings.

***Model 4 (exploratory correlated factors)***

Inspired by our multidimensional model (Model 2), Model 4 explored whether our aggregated data could be represented by three correlated latent factors using “oblimin” rotation. Compared to the confirmatory models, Model 4 explained more total item variance (56.21%) and exhibited acceptable model fit for CFI = .958, TLI = .922, and RMSEA (90% *CI*) = .077 (.059−.096), and improved relative model fit, BIC = – 127. As illustrated by Table SM-4, the factor loading patterns of two latent factors diverged from CHE’s proposed structure.

The first factor “successful and connected” received main loadings ≥ .30 from three goal-directed action facets (successful outcomes, environmental support, positive affect) and two momentary experience facets (connection, fulfilment). Conversely, the second factor “engaging mentally” received main loadings ≥ .30 from two momentary experience facets (engagement, mindfulness) and two goal-directed action facets (cognitive skills, coping and action). The loading patterns suggests that Model 4 organizes CHE’s state-level facets by their emphasis on external vs. internal processes rather than organizing them into goal pursuit vs. experiencing the moment. Nevertheless, the distinction between internal and external processes is not clearcut because “engaging mentally” received a meaningful cross-loading from interpersonal skills.

In contrast to the other data-driven factors, “stable qualities” represents its theorized CHE source by receiving main loadings ≥ .30 from internal competencies, wellbeing, resilience, and interpersonal skills. “Stable qualities” also received a meaningful cross-loading from coping and action (.38), perhaps suggesting that individuals routinely apply coping skills in challenging situations.

Overall, “successful and connected” presented with the largest mean factor loading and proportion of variance explained (*λ* = .75, R^2^ = 42.24%) followed by “stable qualities” (*λ* = .65, R^2^ = 29.96%) and “engaging mentally” (*λ* = .60, R^2^ = 27.80%) and. In contrast to Model 2, the inter-factor correlations in Model 4 ranged from .41 to .58, suggesting a conceptually interpretable solution. We next explored whether adding a general factor may further improve model fit, explained variance, and conceptual interpretability.

**Table SM-4**

*Model 4 (exploratory correlated factors)*

| **Source** | **Facet** | **Successful / connected *(𝛌)*** | **Engaging mentally *(𝛌)*** | **Stable qualities *(𝛌)*** | ***𝛅*** |
| --- | --- | --- | --- | --- | --- |
| Goal | Successful outcomes | **.83** | –.01 | .00 | .32 |
| Moment | Connection | **.79** | .12 | –.11 | .39 |
| Moment | Fulfilment | **.73** | –.01 | .16 | .32 |
| Goal | Environmental support | **.72** | –.02 | –.01 | .50 |
| Goal | Positive affect | **.70** | –.02 | .18 | .35 |
| Moment | Engagement | .12 | **.75** | –.08 | .37 |
| Goal | Cognitive skills | .11 | **.62** | .15 | .41 |
| Moment | Mindfulness | .07 | **.61** | .07 | .52 |
| Goal | Coping and action | –.07 | **.43** | **.38** | .57 |
| Qual | Internal competencies | .09 | –.07 | **.88** | .20 |
| Qual | Wellbeing | .04 | .03 | **.64** | .55 |
| Qual | Resilience | –.07 | .13 | **.61** | .59 |
| Qual | Interpersonal skills | –.09 | **.30** | **.48** | .61 |

*Note.* Exploratory three-factor solution with “oblimin” rotation organizing the 13 CHE facets. The factor “successful and connected” received main loadings from five facets; “engaging mentally” and “stable qualities” received main loadings from four facets each. Model 4 accounted for 56.21% of total variance with the factors contributing at the following proportions: “successful and connected” explained 42.24%, “engaging mentally” 27.80%, and “stable qualities” 29.96%. Factor scores ≥ .30 are bolded. Goal = goal-directed action; Moment = momentary experience; Qual = stable qualities. *λ* = factor loading; *δ* = uniqueness. *N* = 251

***Model 5 (exploratory bifactor) – reported in the main text***

Because our confirmatory analyses indicated that a bifactor model may best represent our data set, we established a final exploratory factor model including a general latent factor in addition to three orthogonal group factors (resembling Model 3). Compared to all other models, Model 5 explained most total item variance (58.98%), exhibited the best absolute model fit, CFI = .971, TLI = .928, and RMSEA (90% *CI*) = .074 (.053−.096), and the second-best relative model fit, BIC = −101. Model 5 also provides a compelling conceptual interpretation that supports observations of a dominant general eustress factor from Model 3 and previous research (Kloidt & Barsalou, 2025). For these reasons, we report Model 5 in the main text.

Table SM-5 illustrates that the “general eustress” factor received meaningful loadings > .30 from all facets, including strongest loadings from successful outcomes (.80), positive affect (.79), and fulfilment (.78). The general factor had a mean factor loading of .60 and accounted for 64.98% of the explained variance, demonstrating that the 13 CHE facets captured a dominant, general construct of eustress.

The group factors from Model 5 closely resemble the correlated data-driven factors from Model 4. The group factor “stable qualities” represents its respective CHE source by receiving main loadings ≥ .30 from internal competencies, wellbeing, resilience, and interpersonal skills. As in Model 4, “stable qualities” further received a meaningful cross-loading from coping and action (.38). For “stable qualities”, the average absolute main loading was .53 and the factor accounted for 17.59% of variance explained.

The second group factor “engaging mentally” received main loadings ≥ |.30| from two momentary experience facets and three goal-directed action facets, with the main loading for successful outcomes being negative (−.31). Although negative loadings may limit the interpretability of a latent factor, this specific negative loading can be interpreted as an emphasis on mental skills and effort required in a challenging situation over anticipating its desirable outcomes. Overall, “engaging mentally” had an average absolute main loading of .34 and accounted for 8.80% of explained variance.

The third group factor “feeling supported and connected” received two sizeable main loadings ≥ .30 (environmental support, connection) and two negligible main loadings < .30 (fulfilment, positive affect). Whereas latent factors with few strongly loading items may indicate an unidentified and potentially unstable factor solution, these findings are more common for bifactor models where group factors capture residual variance in the facets after the general factor first accounted for common variance across all facets. Despite two negligible main loadings, the average main loading was meaningful (.34) and the factor accounted for 8.62% of explained variance. For these reasons we selected Model 5 as the best-fitting and conceptually interpretable solution to be presented in the main text.

**Table SM-5**

*Model 5 (exploratory bifactor)*

| **Source** | **Facet** | **General eustress *(𝛌)*** | **Stable qualities *(𝛌)*** | **Engaging mentally *(𝛌)*** | **Feeling supported / connected *(𝛌)*** | ***𝛅*** |
| --- | --- | --- | --- | --- | --- | --- |
| Qual | Internal competencies | **.55** | **.71** | –.07 | –.03 | .19 |
| Qual | Wellbeing | **.40** | **.54** | .09 | .11 | .53 |
| Qual | Resilience | **.41** | **.48** | .05 | –.13 | .58 |
| Qual | Interpersonal skills | **.45** | **.38** | .18 | –.11 | .61 |
| Moment | Engagement | **.68** | –.12 | **.40** | –.05 | .37 |
| Goal | Cognitive skills | **.68** | .07 | **.34** | –.03 | .42 |
| Moment | Mindfulness | **.60** | .02 | **.34** | –.03 | .52 |
| Goal | Coping and action | **.49** | **.32** | **.33** | .01 | .55 |
| Goal | Successful outcomes | **.80** | –.15 | **–.31** | .13 | .22 |
| Goal | Environmental support | **.56** | –.06 | .00 | **.61** | .31 |
| Moment | Connection | **.65** | .03 | –.14 | **.42** | .38 |
| Moment | Fulfilment | **.78** | –.11 | –.08 | .19 | .33 |
| Goal | Positive affect | **.79** | –.13 | –.10 | .14 | .33 |

*Note.* Exploratory bifactor solution with orthogonal rotation organizing the 13 CHE facets. The general factor includes all CHE facets. The group factors “stable qualities” and “feeling supported and connected” received main loadings from four facets each; “engaging mentally” received main loadings from five facets. Model 5 accounted for 58.98% of total variance with the factors contributing at the following proportions: “general eustress” explained 64.98%; “stable qualities” explained 17.59%, “engaging mentally” 8.80%, and “feeling supported and connected” 8.62%. Factor scores ≥ |.30| are bolded. Goal = goal-directed action; Moment = momentary experience; Qual = stable qualities. *λ* = factor loading; *δ* = uniqueness. *N* = 251

**References**

Brown, T. A. (2015). *Confirmatory factor analysis for applied research* (2nd edn). Guilford Press.

Kloidt, J., & Barsalou, L. W. (2025). *Developing and evaluating the Comprehensive Hierarchical Eustress Review (CHER)*. PsyArXiv. https://doi.org/10.31234/osf.io/3wmhs_v1
